# Models of Care of Schizophrenia in the Community—An International Perspective

**DOI:** 10.1007/s11920-022-01329-0

**Published:** 2022-03-01

**Authors:** Guru S. Gowda, Mohan K. Isaac

**Affiliations:** 1 Department of Psychiatry, National Institute of Mental Health and Neuro Sciences (NIMHANS), Bengaluru, 560029 India; 2 Clinical Professor of Psychiatry, Division of Psychiatry, Faculty of Health and Medical Sciences, The University of Western Australia, Fremantle Hospital, Fremantle Hospital, Level 7, T Block, Fremantle, WA 6160 Australia

**Keywords:** Schizophrenia, Disability, Schizophrenia/rehabilitation, Community care

## Abstract

**Purpose of Review:**

We reviewed the existing and recent community models of care in schizophrenia. We examine characteristics, recent updates, evidence, cost-effectiveness, and patients’ acceptance for existing and new community-based care models in high-income (HI) and low- and middle-income (LAMI) countries.

**Recent Findings:**

Assertive Community Treatment (ACT), Intensive Case Management (ICM), and Crisis Intervention are cost-effective interventions for schizophrenia and time tested in the last few decades in HI countries. The growing evidence suggests that tailor-made ACTs and ICM can effectively reduce substance use, homelessness, and criminal activity in persons with schizophrenia who live in the community. Similarly, in LAMI Countries, a few community-based care models for schizophrenia have been developed and tested based on community-based rehabilitation principles.

**Summary:**

The modality of a community model of care and interventions for a person with schizophrenia should be chosen based on the person’s co-existing psychosocial difficulties and challenges such as homelessness, criminal behaviour, and substance use.

**Supplementary Information:**

The online version contains supplementary material available at 10.1007/s11920-022-01329-0.

## Introduction

Schizophrenia has a chronic and debilitating course, usually marked by several relapses. This disorder is characterized by persistent cognitive impairment and positive and negative symptoms. Despite a century of research, the disorder’s genesis, aetiology, and prevention of relapse remain a mystery [1], and current pharmacological interventions are only modestly effective [2]. Due to varying degrees of functional and social impairment in schizophrenia, despite adequate antipsychotic treatment, individuals struggle to maintain their employment, social relationships, and ability to live independently [3, 4]. Relapse in the symptoms of schizophrenia could be due to poor medication adherence or the presence of ongoing or emergence of psychosocial stressors. These elements make the course of the illness chronic and phasic [5]. In addition, the relapse of schizophrenia symptoms is associated with increased re-admission, emergency care visits, and increased healthcare resource utilization [6]. As per the recent systematic analysis for the Global Burden of Disease Study 2017, schizophrenia is one of the world’s top 20 leading causes of years lived with disability [7•], and 70%–90% of persons with schizophrenia have employment and housing difficulty [4].

Given the magnitude of the problem, the World Health Organization’s Mental Health Action Plan and the World Psychiatric Association emphasized community-based mental health services and interventional programmes [8, 9, 10•, 11]. This was intended to integrate mental health into the community, identify alternate solutions such as deinstitutionalizing asylums and mental hospitals worldwide, and promote mental health by improving accessibility, acceptability, affordability, availability, and scalability.

Community-based interventional programmes include Case Management (CM), Intensive Case Management (ICM), Crisis Intervention (CI), and Assertive Community Treatments (ACT). In addition, emphasis was placed on supported housing for people with schizophrenia who are at high risk of homelessness or is homeless. The existing supported housing models include the Clubhouse model, Housing first, Home Again and Clustered Group Homes. These were developed as a potential approach to provide recovery-oriented, evidence-based care in the community and provide long-term care for a person with severe mental illness (SMI) like Schizophrenia [12]. It is thought to reduce the significant human and economic costs associated with schizophrenia relapse and readmission; however, the intervention's impact showed mixed results [2]. This review addresses the characteristics and recent updates on existing and new community-based care models for schizophrenia worldwide.

## Community-based Interventional Programmes

### Case Management

Case management (CM) is an approach to coordinating community-based integrated health and social care services. This model of care emerged as a response to the deinstitutionalization of psychiatric care in the 1960s. It facilitates the horizontal integration of care for SMIs across health and social services. Case managers will try to understand the client’s needs, develop a care plan, connect them to the services they need, and assist patients in maintaining regular engagement with psychiatric services [13]. The case manager may be a registered psychiatric nurse, a social worker, or an occupational therapist [14]. The model will be based on the assumption that everyone, regardless of SMI, can use, develop, and utilize existing services with support and guidance. The reviews on CM show that it was significantly more effective than standard outpatient care in providing community-based care for patients with SMIs, particularly in ensuring continuity of care [2].

Despite expressed needs from the persons with schizophrenia and their caregivers on CM during focused group discussion (14), this model did not reportedly impact the overall improvement of patients’ clinical condition or readmission rate into the hospital. As a result, there was no significant impact on healthcare costs [2, 15]. It was also found that the CM model of care was not only used for community management of psychotic disorder treatment in remote rural areas [16] but was also adapted for substance use disorder management in the community [17] and those at risk of homelessness with SMI [18••]. Furthermore, the recent systemic review by Ponka et al. suggests that the CM is beneficial and effective when case managers have low caseloads and high intensity of direct continuity of care, especially when dealing with SMI. The latter have complex psychosocial needs such as housing stability, unemployment, and comorbid substance use disorder [18••]. Thus, the overall benefits of CM, such as client acceptance and satisfaction and its effectiveness in ensuring continuity of care, may outweigh the costs of healthcare and re-hospitalization.

## Intensive Case Management

Intensive Case Management (ICM) is a model of care, where it provides care to small caseloads (fewer than 20). ICM interventions are of high-intensity input, such as providing 24-h emergency care, with a particular focus on medication compliance, and practising ‘assertive outreach’ on uncooperative clients either at home or workplace. ICM care is provided by a multidisciplinary team of medical and paramedical professionals (nurse practitioners, psychiatrists, outreach social workers, and others) or by case managers with varied educational backgrounds. The ICM model has lost some originality and richness due to its similarity to the original model of ACT and CM; however, ICM services are more intensive than CM and more flexible than ACT services [19]. The ICM model of care was used for community management of schizophrenia and was also helpful in managing comorbid problematic substance use (alcohol) and homelessness with SMI in the community. According to the initial systematic review and meta-regression, ICM is suitable for individuals with SMI living in the community who frequently require hospitalization [20]. A recent review indicates, ICM interventions may improve the psychological symptoms, quality of life, and social functioning, reduce problematic alcohol use, decrease hospitalization, increase care retention, and reduce emergency hospital visits when compared to standard case management or usual services, but provided mixed results on housing stability, with fewer days spent homeless and lower unemployment rates [18••, 21••]. ICM is better than other models due to its more intensive direct continuous care and flexible approach. Furthermore, compared to ACT or CM services, the service provides a more client-friendly, acceptable, and satisfied model of care in the community for SMI.

## Assertive Community Treatment

Assertive Community Treatment (ACT) is a team-based approach with low caseload and high-intensity services such as providing 24-h emergency care, focusing on medication compliance and engagement in “assertive outreach” uncooperative clients. Furthermore, ACT directly provides all necessary care at home or work, unlike CM, which coordinates with the other service providers. The ACT team includes social workers, nurses, and psychiatrists, catering exclusively to a defined group of patients and works as a multidisciplinary team [21••]. The ACT team provides holistic care to the patient in the community by assisting with illness management, medication management, housing, finances, and daily living needs such as shopping and public transportation. It is believed to be a structured, evidence-based, practical, and cost-effective community-based recovery-oriented model of care for schizophrenia and other SMIs [22]. It is observed that the ACT model of care is not only used for the community care of schizophrenia and other psychotic disorders [22] but also adapted and customized for the care of persons who are homeless [23] in the management of substance use disorders like alcohol [24] and persons with psychotic disorders with legal issues [25, 26•]. The existing randomized controlled studies [24, 25, 26•] and meta-analysis [23] done over the last two decades have shown effectiveness in reducing the mean length of stay in the hospital, reducing re-hospitalization and cost-effectiveness in schizophrenia and other SMIs compared to the standard of care or CM model [22]. In addition, customized ACTs effectively reduce substance use, homelessness [23, 27], and criminal activity in persons with psychotic disorders living in the community [25, 26•]. The Netherlands and Hamburg have adopted similar ACT models, such as the Flexible Assertive Community Treatment model and the Therapeutic Assertive Community Treatment model in the community [28]. The ACT model is more effective than the standard of care or CM; studies on clients and community groups have also shown a high level of satisfaction with the ACT services due to their holistic and recovery-oriented approach [29, 30]. However, it has been criticized from the patient's rights perspective for compromising individual autonomy, independent living and for being coercive [31]. The overall benefits, including clients’ satisfaction, may be worth the perceived limitation in patient autonomy.

## Crisis Intervention

The Crisis Intervention (CI) model was developed to provide intervention during a psychiatric crisis in the community. It was assumed that people with schizophrenia and other SMIs would have poor coping mechanisms and are likely to decompensate in response to severe stress. This could lead to symptom exacerbation or might lead to a relapse of the illness. In this model, trained personnel provide round-the-clock interventions. Following the crisis, the professional providers or the Crisis Resolution and Home Treatment (CRHT) teams will assess the clients and identify the issues [32]. Subsequently, the team will assist the client in dealing with the crisis by providing practical guidance in living skills, supporting family members, counselling, and treatment rationalization. The CI team comprises multidisciplinary specialists and members who have been specially trained in Crisis Intervention methods, including the unique sociocultural and community needs of the person with SMI. As a community-based mobile team, the CI team provides access to hospital care, day care, or residential care to clients in crisis. Countries like Australia, the UK, and the USA have adopted the CI approach as a model in the community care of SMI. This model reduces hospitalizations and relapse of symptoms [2]. The Cochrane review endorses the CI model as a viable and acceptable model for SMI in the community [33]. The CI model is superior to standard care in reducing readmissions and family burden, reducing the perceived stigma and cost-effectiveness but has no impact on the mortality associated with SMI [34–36].

Furthermore, patients and relatives are more satisfied with the CI care model than with the standard care model when providing home or community-based care [37]. Unfortunately, few countries have developed a joint crisis plan in recent years. In such a plan, the client and clinician collaborate to have advance agreements for the anticipated crisis due to mental illness. This, in turn, allows clients to feel more independent and in control of their care in the event of a future crisis while incapacitated.

## Community Care for People with Schizophrenia in Low- and Middle-Income Countries (LAMICs)

 Due to poor resource allocation and a lack of human resources, community care services for schizophrenia in low- and middle-income countries face challenges. To address this, LAMICs have developed a few community-based research models for SMI, such as schizophrenia, which adopt the community-based rehabilitation (CBR) strategy and provide community care by appropriately trained community health workers. These include ***collaborative community-based care (CCBC) intervention***, in which trained CHWs provide structured psychoeducational information, medication adherence management, health promotion, specific rehabilitation needs, and linking with self-help groups and networks with community agencies. The CCBC intervention is effective and cost-effective compared to commonly available care [38]. The same research group developed **Community care for People with Schizophrenia in India (COPSI)’;** this model, similar to the above, provides structured psychoeducational information, adherence management strategies, specific rehabilitation strategies, linkage to self-help groups, and networks with community agencies for people with schizophrenia.

Such community intervention is provided by trained CHWs over 12 months and supervised by Intervention Coordinators (Psychiatric Social Workers) (COPSI). This intervention is modestly more effective than facility-based care, particularly reducing disability and psychotic symptoms [39]. Later, the same group of researchers attempted to examine the effectiveness of community service delivery by trained lay health workers supervised by specialists. The results were promising, indicating an acceptable and feasible intervention for treating schizophrenia in India [40]. In China, studies on psychoeducational family intervention focusing on patients’ recognition of illness, caring attitude, treatment compliance, relapse prevention, and social functioning were observed to be effective and appropriate for psychiatric rehabilitation in rural communities [41–43]. The research team approached the community 14 years later to assess its long-term effectiveness. Patients’ treatment adherence/compliance and social functioning improved in the intervention arm [44]. In Ethiopia, district-level mental health care was integrated into primary care, and improvements in clinical and social outcomes of SMI and better human resource allocation in the community were observed [45].

Furthermore, researchers examined Community-based Rehabilitation Intervention for People with Schizophrenia in Ethiopia (RISE), which used a community-based rehabilitation (CBR) strategy and provided community care. They found that the results were promising in improving disability outcomes [46]. As part of the aftercare services for SMI, researchers in Iran provided treatment follow-up, family psychoeducation, and patient social skills training to people with schizophrenia and other psychotic disorders. After 20 months of intervention, services were more effective than the control arm at reducing the need for re-hospitalization and the severity of psychopathology [47]. In recent years, various non-governmental organizations have provided comprehensive psychosocial care with trained laypeople or community health workers collaborating with state governments. The Banyan Rural mental health programme, BasicNeeds-UK (Jharkhand), VOLCOMH outreach programme (Mizoram), The Chellamuthu Trust vocational rehabilitation units for the mentally ill (Tamil Nadu), Taluk Mental Health Programme, and part of Care at Door-steps for people with severe mental disorders are a few examples (Karnataka district mental health programme) [48, 49•, 50•]. Even though CBR models for schizophrenia care were acceptable, feasible, and effective in the community [51•] and could be delivered by a non-specialist like a trained layperson or a trained community health worker [52], they were not replicated or scaled up to the expected extent in LAMICs to increase coverage and improve outcomes for people with schizophrenia and their caregivers. In addition, due to the complexity and challenge of researching low-and middle-income countries, assessing the economic effectiveness of CBR for people with schizophrenia is difficult [53•]. The degree to which community care interventions components were integrated with primary care services varied significantly within and across LAMICs [10•]. The effective use of community service could be used as an indicator of the severity of mental illness in the community [54] and the prevalence of Homelessness in Persons with Mental Illness (HPMI).

## Supported Housing Community Models

Globally, in addition to the community care model of care for SMI, emphasis was placed on supported housing and employment for SMI for homeless individuals or those at high risk for homelessness and/or not having stable employment. These approaches were developed to provide responsible alternatives for long-term care and to promote community living and engagement. Some of the existing supported housing models to facilitate SMI living in the community are described below.

## Clubhouse Model

The clubhouse model is a psychosocial rehabilitation model utilized for more than 70 years. The Clubhouse comprises non-clinical, integrated therapeutic working communities for people with SMI. The model’s concept is based on empowerment of people with SMI and facilitating ‘peer-help’. It has four guiding principles: a) a place to go to; b) meaningful work; c) meaningful relationships; and d) a place meet. Persons with SMI can develop a sense of community through a safe environment, supportive relationships, and clubhouse employment activities. Furthermore, the members of the clubhouse share ownership and responsibility for the Clubhouse’s success, which aids in developing a purpose and goals. The clubhouse model, estimated to cost one-third of inpatient services, promotes employment, improves the quality of life, and reduces re-hospitalization rates [55, 56•].

## Housing First

Housing First is an evidence-based practice model currently used by the USA and Canadian governments to address homelessness among people with SMI. It is regarded as a non-coercive model for homeless persons in the community. Systematic reviews and randomized controlled trials have found that change this sentence to Housing First is an effective strategy for people with SMI, who are at risk of homelessness, in improving their quality of life and community functioning and in reducing the rate of homelessness in the community [57, 58•]. Unlike many high-income countries, LAMIC lacks tailor-made, specific legislation or acts for homelessness. However, the NGOs sector has taken the lead, mainly providing care for those with SMI and homelessness, using a concept of community-based inclusive living. The NGOs’ common objective is to a) identify and admit persons with SMI into their centres, b) treat their mental and physical health impairments, and c) make efforts to rehabilitate and reintegrate them into their communities and families using available local resources. If they cannot reintegrate into the community, they will be housed in the NGO provided housing [59–62].

## Home Again (HA)

The Home Again model, based on the patient’s choice, provides housing and community-based rehabilitation services. In each house, the shelter will be provided to three to five people with SMIs who are clinically stable and require community care due to moderate disability. It will be managed by a trained onsite personal assistant (social worker) who will visit or live with them and assist with services tailored to their needs. Such ‘Home Again’ groups are overseen by a multidisciplinary team comprising a programme manager, a case manager, and a nurse. The person with SMI in ‘House Again’ can take care of all self-care needs such as cooking, housekeeping, purchase of monthly supplies, etc. Furthermore, individuals are engaged in flexible work opportunities based on their abilities. Community engagement is encouraged through a monthly incentive that provides an opportunity to open local bank accounts and conduct banking transactions. A social worker will coordinate treatment with a multidisciplinary team. Those who eventually become independent, no longer require supervision from a personal assistant, and have a less social and occupational disability get promoted to ‘Independent Living’. The individual will continue to receive outpatient clinical and social care from the multidisciplinary team in such a case. This framework was designed by ‘Banyan’, a mental health organization in Chennai, Tamil Nadu, India, with support from national and international organizations. The ‘Banyan’ home is based on a continuum of care model, with a single-staged transitional housing model followed by independent housing [63, 64]. Similar interventions have been carried out in Kerala’s Malappuram and Thrissur and Assam’s Guwahati and Boko, collaborating with Ashadeep (an NGO) in India. Compared to a matched group of people who remain in institutional settings, people in HA have higher levels of community integration and lower levels of disability [65]. This model upholds individual autonomy, the right to community living and identity, employment and security without discrimination in the community, consistent with the Mental Health Care Act, 2017 [66].

## Clustered Group Homes

A Clustered Group Home is a quasi-institutional psychiatric facility for moderate to severe mental illnesses requiring inpatient care. Such a facility will have eight cottages, six to eight people each house. In the clustered cottages, a total of 45–50 patients live together. A multidisciplinary team of personal assistants, nursing staff, social workers, clinical psychologists, and a psychiatrist looks after the individual’s well-being and adapts to a quasi-institutional psychiatric ecosystem [63, 64]. Over time, those who improved in regaining their independent living skills needed reduced supervision and less social and occupational disability were supported by moving into either ‘Home Again’ or ‘Independent Living’ models. This framework was designed by the ‘Banyan’, based on the supportive housing model elements. This model empowers individual autonomy and upholds the right to community living advocated in Mental Health Care Act, 2017 [66]. Even though many programmes and different supported-housing models exist in LAMICs for homelessness with SMI, there is a need for rigorous evaluation to identify critical aspects required for individuals to achieve long-term recovery and cost-effectiveness [67].

## Conclusion

Community care models are developed using the personal recovery principle and quality of life in persons with schizophrenia and SMI. In high-income countries, interventions such as Assertive Community Treatment, Intensive Case Management, and Crisis Intervention have been found cost-effective and time-tested in the last few decades. The modality of interventions chosen is based on the person’s co-existing psychosocial difficulties and challenges. Similarly, in LAMICs, community-based care based on the CBR principle has been developed and tested in the community. It can be provided by a ‘lay person’ to ‘non-professional’. These models include collaborative community-based care (CCBC) interventions, Community care for People with Schizophrenia in India (COPSI) and Community-based Rehabilitation Intervention for People with Schizophrenia in Ethiopia (RISE). However, they were not replicated or scaled up to the extent expected in LAMIC countries. Except for the ‘clubhouse model’ and ‘housing first’ supported housing models, there is little research on Home Again and Clustered Group Homes models on long-term recovery and cost-effectiveness in the community in LAMICs. There is a significant variation in the supported housing model of care and services within and across LAMICs. There is a need for rigorous evaluation of long-term recovery and cost-effectiveness.

## Supplementary Information

Below is the link to the electronic supplementary material.

Supplementary file1 (PDF 1225 KB)

Supplementary file2 (PDF 1225 KB)

## References

[1] Tandon R, Nasrallah HA, Keshavan MS (2009). Schizophrenia, "just the facts" 4. Clinical features and conceptualization Schizophr Res.

[2] Adamou M (2005). Community service models for schizophrenia: evidence-based implications and future directions. Psychiatry (Edgmont).

[3] Tandon R, Keshavan MS, Nasrallah HA (2008). Schizophrenia, "Just the Facts": what we know in 2008 part 1: overview. Schizophr Res.

[4] Harvey PD, Strassnig MT, Silberstein J. Prediction of disability in schizophrenia: Symptoms, cognition, and self-assessment. Journal of Experimental Psychopathology. 2019;10(3).

[5] Higashi K, Medic G, Littlewood KJ, Diez T, Granström O, De Hert M (2013). Medication adherence in schizophrenia: factors influencing adherence and consequences of nonadherence, a systematic literature review. Ther Adv Psychopharmacol.

[6] Gowda GS, Das S, Nanjegowda RB (2017). Psychotropic implant can be a new hope in psychiatry. Asian J Psychiatr.

[7] • GBD 2017 Disease and Injury Incidence and Prevalence Collaborators (2018). Global, regional, and national incidence, prevalence, and years lived with disability for 354 diseases and injuries for 195 countries and territories, 1990-2017: a systematic analysis for the Global Burden of Disease Study 2017. Lancet (London, England), 392(10159): 1789–1858. This paper provides an update and a global picture of years lived with disability for various leading diseases, besides their incidence and prevalence. 10.1016/S0140-6736(18)32279-7 PMC6227754 30496104

[8] WHO. Mental Health Action Plan 2013–2020. World Health Organization; Geneva, Switzerland: 2013. Available at https://www.who.int/publications/i/item/9789241506021.

[9] Castillo EG, Ijadi-Maghsoodi R, Shadravan S, Moore E, Mensah MO, Docherty M (2019). Community Interventions to Promote Mental Health and Social Equity. Curr Psychiatry Rep.

[10] Kohrt BA, Asher L, Bhardwaj A, Fazel M, Jordans MJD, Mutamba BB (2018). The Role of Communities in Mental Health Care in Low- and Middle-Income Countries: A Meta-Review of Components and Competencies. Int J Environ Res Public Health.

[11] Thornicroft G, Alem A, Antunes Dos Santos R, Barley E, Drake RE, Gregorio G, et al. WPA guidance on steps, obstacles and mistakes to avoid in the implementation of community mental health care. World Psychiatry. 2010;9(2):67–77. 10.1002/j.2051-5545.2010.tb00276.x PMC2911080 20671888

[12] Thornicroft G, Deb T, Henderson C (2016). Community mental health care worldwide: current status and further developments. World Psychiatry.

[13] Lukersmith S, Millington M, Salvador-Carulla L (2016). What Is Case Management? A Scoping and Mapping Review. Int J Integr Care.

[14] Wong HH, Yong YH, Shahwan S, Cetty L, Vaingankar J, Hon C (2019). Case management in early psychosis intervention programme: Perspectives of clients and caregivers. Early Interv Psychiatry.

[15] Holloway F (1991). Case management for the mentally ill: Looking at the evidence. Int J Soc Psychiatry.

[16] Gong W, Xu D, Zhou L, Brown HS, Smith KL, Xiao S (2014). Village doctor-assisted case management of rural patients with schizophrenia: protocol for a cluster randomized control trial. Implement Sci.

[17] Vanderplasschen W, Rapp RC, De Maeyer J, Van Den Noortgate W (2019). A Meta-Analysis of the Efficacy of Case Management for Substance Use Disorders: A Recovery Perspective. Front Psychiatry.

[18] •• Ponka D, Agbata E, Kendall C, Stergiopoulos V, Mendonca O, Magwood O, et al. The effectiveness of case management interventions for the homeless, vulnerably housed and persons with lived experience: A systematic review. PLoS One. 2020;15(4):e0230896. **This is a recent well conducted comprehensive review of effectiveness of case management interventions in the community for vulnerable population.** 10.1371/journal.pone.0230896 PMC7313544 32271769

[19] Drukker M, Maarschalkerweerd M, Bak M, Driessen G, a Campo J, de Bie A, et al. A real‐life observational study of the effectiveness of FACT in a Dutch mental health region. BMC Psychiatry. 2008;8:93. 10.1186/1471-244X-8-93 PMC2629765 19055813

[20] Burns T, Catty J, Dash M, Roberts C, Lockwood A, Marshall M (2007). Use of intensive case management to reduce time in hospital in people with severe mental illness: systematic review and meta-regression. BMJ.

[21] •• Dieterich M, Irving CB, Park B, Marshall M. Intensive case management for severe mental illness. Cochrane Database Syst Rev. 2010;(10):CD007906. 10.1002/14651858.CD007906.pub2. Update in: Cochrane Database Syst Rev. 2017 Jan 06;1. **A****recent comprehensive Cochrane review on effectiveness of Intensive case management for severe mental illness which shows that intensive care management may reduce hospitalization, increase retention in care and also globally improved social functioning.** 10.1002/14651858.CD007906.pub3 PMC6472672 28067944

[22] Bond GR, Drake RE (2015). The critical ingredients of assertive community treatment. World Psychiatry.

[23] Coldwell CM, Bender WS (2007). The effectiveness of assertive community treatment for homeless populations with severe mental illness: a meta-analysis. Am J Psychiatry.

[24] Gilburt H, Burns T, Copello A, Coulton S, Crawford M, Day E (2012). Assertive Community Treatment for alcohol dependence (ACTAD): study protocol for a randomized controlled trial. Trials.

[25] Cusack KJ, Morrissey JP, Cuddeback GS, Prins A, Williams DM (2010). Criminal justice involvement, behavioral health service use, and costs of forensic assertive community treatment: a randomized trial. Community Ment Health J.

[26] • Lamberti JS, Weisman RL, Cerulli C, Williams GC, Jacobowitz DB, Mueser KT, et al. A Randomized Controlled Trial of the Rochester Forensic Assertive Community Treatment Model. Psychiatr Serv. 2017;68(10):1016–1024. **A****randomised controlled trial on effectiveness Forensic Assertive Community Treatment Model to reduce the criminal behaviour in persons with SMI**. 10.1176/appi.ps.201600329 PMC7369621 28566028

[27] Morse GA, Calsyn RJ, Klinkenberg WD, Trusty ML, Gerber F, Smith R, Tempelhoff B, Ahmad L (1997). An experimental comparison of three types of case management for homeless mentally ill persons. Psychiatr Serv.

[28] Lambert M, Kraft V, Rohenkohl A, Ruppelt F, Schröter R, Lüdecke D, et al. Innovative Versorgungsmodelle für Menschen mit schizophrenen Erkrankungen [Innovative care models for people with schizophrenia]. Bundesgesundheitsblatt Gesundheitsforschung Gesundheitsschutz. 2019;62(2):163–172. German. 10.1007/s00103-018-2868-y 30623205

[29] Chue P, Tibbo P, Wright E, Van Ens J (2004). Client and community services satisfaction with an assertive community treatment subprogram for inner-city clients in Edmonton. Alberta Can J Psychiatry.

[30] Lofthus AM, Westerlund H, Bjørgen D, Lindstrøm JC, Lauveng A, Clausen H (2016). Are Users Satisfied with Assertive Community Treatment in Spite of Personal Restrictions?. Community Ment Health J.

[31] Lamberti JS, Russ A, Cerulli C, Weisman RL, Jacobowitz D, Williams GC (2014). Patient experiences of autonomy and coercion while receiving legal leverage in forensic assertive community treatment. Harv Rev Psychiatry.

[32] Stevens L, Rodin I. Mental health services II, Psychiatry (Second Edition), Churchill Livingstone, 2011, Pages 4–5, ISBN 9780702033964. 10.1016/B978-0-7020-3396-4.00006-8. (https://www.sciencedirect.com/science/article/pii/B9780702033964000068). [Assessed 3rd June 2021].

[33] Joy CB, Adams CE, Rice K. Crisis intervention for people with severe mental illnesses. Cochrane Database of Systematic Reviews 2006;4. 10.1002/14651858.CD001087.pub3. 10.1002/14651858.CD001087.pub3 17054133

[34] Johnson S, Nolan F, Pilling S, Sandor A, Hoult J, McKenzie N (2005). Randomised controlled trial of acute mental health care by a crisis resolution team: the north Islington crisis study. BMJ.

[35] Murphy S, Irving CB, Adams CE, Driver R. Crisis intervention for people with severe mental illnesses. Cochrane Database Syst Rev. 2012;5(5). 10.1002/14651858.CD001087.pub4 PMC4204394 22592673

[36] Fenton WS, Hoch JS, Herrell JM, Mosher L, Dixon L (2002). Cost and cost-effectiveness of hospital vs residential crisis care for patients who have serious mental illness. Arch Gen Psychiatry.

[37] Howard L, Flach C, Leese M, Byford S, Killaspy H, Cole L (2010). effectiveness and cost-effectiveness of admissions to women's crisis houses compared with traditional psychiatric wards: pilot patient-preference randomized controlled trial. British J Psychiatry.

[38] Chatterjee S, Leese M, Koschorke M, McCrone P, Naik S, John S (2011). Collaborative community based care for people and their families living with schizophrenia in India: protocol for a randomised controlled trial. Trials.

[39] Chatterjee S, Naik S, John S, Dabholkar H, Balaji M, Koschorke M (2014). Effectiveness of a community-based intervention for people with schizophrenia and their caregivers in India (COPSI): a randomised controlled trial. Lancet.

[40] Balaji M, Chatterjee S, Koschorke M, Rangaswamy T, Chavan A, Dabholkar H (2012). The development of a lay health worker delivered collaborative community based intervention for people with schizophrenia in India. BMC Health Serv Res.

[41] Li Z, Arthur D (2005). Family education for people with schizophrenia in Beijing, China: randomised controlled trial. Br J Psychiatry.

[42] Ran MS, Xiang MZ, Chan CL, Leff J, Simpson P, Huang MS (2003). Effectiveness of psychoeducational intervention for rural Chinese families experiencing schizophrenia–a randomised controlled trial. Soc Psychiatry Psychiatr Epidemiol.

[43] Cai J, Zhu Y, Zhang W, Wang Y, Zhang C (2015). Comprehensive family therapy: an effective approach for cognitive rehabilitation in schizophrenia. Neuropsychiatr Dis Treat.

[44] Ran MS, Chan CL, Ng SM, Guo LT, Xiang MZ (2015). The effectiveness of psychoeducational family intervention for patients with schizophrenia in a 14-year follow-up study in a Chinese rural area. Psychol Med.

[45] Hanlon C, Medhin G, Selamu M, Birhane R, Dewey M, Tirfessa K, et al. Impact of integrated district level mental health care on clinical and social outcomes of people with severe mental illness in rural Ethiopia: an intervention cohort study. Epidemiol Psychiatr Sci. 2019;29:e45. 10.1017/S2045796019000398 PMC8061260 31405401

[46] Asher L, De Silva M, Hanlon C, Weiss HA, Birhane R, Ejigu DA (2016). Community-based Rehabilitation Intervention for people with Schizophrenia in Ethiopia (RISE): study protocol for a cluster randomised controlled trial. Trials.

[47] Ghadiri Vasfi M, Moradi-Lakeh M, Esmaeili N, Soleimani N, Hajebi A (2015). Efficacy of aftercare services for people with severe mental disorders in Iran: a randomized controlled trial. Psychiatr Serv.

[48] van Ginneken N, Maheedhariah MS, Ghani S, Ramakrishna J, Raja A, Patel V. Human resources and models of mental healthcare integration into primary and community care in India: Case studies of 72 programmes. PLoS One. 2017;12(6). 10.1371/journal.pone.0178954 PMC5459474 28582445

[49] • Basavaraju V, Murugesan M, Kumar CN, Gowda GS, Tamaraiselvan SK, Thirthalli J, et al. Care at door-steps for persons with severe mental disorders: A pilot experience from Karnataka district mental health program. Int J Soc Psychiatry. 2020:20764020983856. 10.1177/0020764020983856. (Epub ahead of print). **A recent pilot community intervention strategy for persons with severe mental illness, tested in India.** 10.1177/0020764020983856 33356744

[50] Manjunatha N, Kumar CN, Chander KR, Sadh K, Gowda GS, Vinay B (2019). Taluk Mental Health Program: The new kid on the block?. Indian J Psychiatry.

[51] Asher L, Patel V, De Silva MJ (2017). Community-based psychosocial interventions for people with schizophrenia in low and middle-income countries: systematic review and meta-analysis. BMC Psychiatry.

[52] van Ginneken N, Tharyan P, Lewin S, Rao GN, Meera SM, et al. Non-specialist health worker interventions for the care of mental, neurological and substance-abuse disorders in low- and middle-income countries. Cochrane Database Syst Rev. 2013;(11). 10.1002/14651858.CD009149.pub2 24249541

[53] • Iemmi V, Kumar KS, Blanchet K, Gibson L, Hartley S, Murthy GVS, Patel V, Weber J, Kuper H. Community‐based rehabilitation for people with physical and mental disabilities in low‐ and middle‐income countries. Cochrane Database Syst Rev. 2017;(3):CD010617. **This Cochrane review assessed the effectiveness of community-based rehabilitation (CBR) for people with physical and mental disabilities in low- and middle-income countries.**

[54] Moreno-Küstner B, Mayoral F, Rivas F, Angona P, Requena J, García-Herrera JM (2011). Factors associated with use of community mental health services by schizophrenia patients using multilevel analysis. BMC Health Serv Res.

[55] Bouvet C, Battin C, Le Roy-Hatala C. Le modèle Clubhouse pour les personnes souffrant de troubles psychiques : revue de littérature et expérience française [The Clubhouse model for people with severe mental illnesses: Literature review and French experiment]. Encephale. 2015;41(6):477–86. French. 10.1016/j.encep.2014.09.001 25438970

[56] McKay C, Nugent KL, Johnsen M, Eaton WW, Lidz CW (2018). A Systematic Review of Evidence for the Clubhouse Model of Psychosocial Rehabilitation. Adm Policy Ment Health.

[57] Watson DP, Shuman V, Kowalsky J, Golembiewski E, Brown M (2017). Housing First and harm reduction: a rapid review and document analysis of the US and Canadian open-access literature. Harm Reduct J.

[58] Aubry T, Bourque J, Goering P, Crouse S, Veldhuizen S, LeBlanc S (2019). A randomized controlled trial of the effectiveness of Housing First in a small Canadian City. BMC Public Health.

[59] Gowda GS, Gopika G, Kumar CN, Manjunatha N, Yadav R, Srinivas D (2017). Clinical outcome and rehabilitation of homeless mentally ill patients admitted in mental health institute of South India: "Know the Unknown" project. Asian J Psychiatr.

[60] Gowda GS, Gopika G, Manjunatha N, Kumar CN, Yadav R, Srinivas D (2017). Sociodemographic and clinical profiles of homeless mentally ill admitted in mental health institute of South India: 'Know the Unknown' project. Int J Soc Psychiatry.

[61] Gowda GS, Gopika G, Sanjay TN, Kumar CN, Manjunatha N, Yadav R (2019). Challenges faced by state and society in providing care to homeless mentally Ill Patients: Know the unknown project. Indian J Soc Psychiatry.

[62] Swaminath G (2015). Indian Psychiatric Society-South Zone: Innovations and Challenges in Providing Psychiatric Services to Disadvantaged Populations: A Pilgrim's Progress. Indian J Psychol Med.

[63] Inclusive Living Options | The Banyan- Available on https://thebanyan.org/ilo/#HomeAgain [Last accessed on 2021 Apr 28].

[64] Padmakar A, de Wit EE, Mary S, Regeer E, Bunders-Aelen J, Regeer B. Supported Housing as a recovery option for long-stay patients with severe mental illness in a psychiatric hospital in South India: Learning from an innovative de-hospitalization process. PLoS One. 2020;15(4):e0230074. 10.1371/journal.pone.0230074 PMC7144972 32271784

[65] Narasimhan L, Gopikumar V, Jayakumar V, Bunders J, Regeer B (2019). Responsive mental health systems to address the poverty, homelessness and mental illness nexus: The Banyan experience from India. Int J Ment Health Syst.

[66] Mental Health Care Act. 2017 (MHCA, 2017). Government of India. Available at https://www.prsindia.org/uploads/media/Mental%20Health/Mental%20Healthcare%20Act,%202017.pdf. [Last accessed on 2021 Apr 28].

[67] Smartt C, Prince M, Frissa S, Eaton J, Fekadu A, Hanlon C. Homelessness and severe mental illness in low- and middle-income countries: scoping review. BJPsych Open. 2019;5(4):e57. 10.1192/bjo.2019.32 PMC6611071 31530300

